# Flow-Multi: A Flow-Matching Multi-Reward Framework for Text-to-Image Generation

**DOI:** 10.3390/s26041120

**Published:** 2026-02-09

**Authors:** Jaegun Lee, Janghoon Choi

**Affiliations:** Major in Data Science Convergence, Graduate School of Data Science, Kyungpook National University, Daegu 41566, Republic of Korea

**Keywords:** flow matching, multi-reward reinforcement learning, text-to-image generation

## Abstract

Recent approaches in text-to-image (T2I) generation have actively adopted reinforcement learning (RL) techniques for human preference alignment. However, existing approaches primarily rely on a single reward function, which can lead to overfitting on specific metrics, resulting in issues such as reward hacking and imbalanced optimization among multiple objectives. To address this, we propose Flow-Multi: a flow-matching multi-reward framework for text-to-image generation. Our method builds upon flow-matching-based group-relative policy optimization (GRPO) learning. Each sample is evaluated by four reward models—based on text-to-image alignment, human preference, aesthetic quality, and GenEval—to create a multi-dimensional reward vector. We then utilize the Pareto dominance relationship to remove dominated samples and update the policy using only the non-dominated set. Additionally, we introduce advantage masking during training to suppress the contribution of low-reward samples, ensuring that only high-quality rewards are reflected in policy optimization. Experimental results demonstrate that Flow-Multi achieves balanced improvements across multiple reward criteria compared to the existing Flow-GRPO, validating the effectiveness of the multi-reward reinforcement learning framework for stable alignment in text-to-image generation.

## 1. Introduction

Despite significant advances in text-to-image (T2I) generation [1,2,3], aligning model behavior with human preferences remains a problem with multiple challenges. Beyond mere prompt adherence, users implicitly value several orthogonal qualities—semantic faithfulness, aesthetic appeal, compositional correctness, and robustness on standardized vision–language tests. Optimizing one metric often degrades another, a classic case of “you get what you measure.” As a result, single-reward pipelines tend to overfit, exhibiting reward hacking, distributional brittleness, or drift in unmeasured dimensions.

Recent work explores reinforcement learning (RL) for preference alignment in generative models [4,5,6,7,8], borrowing ideas from reinforcement learning from human feedback (RLHF) and policy optimization [4,9,10]. These approaches typically train with a single scalar reward (e.g., preference score, CLIP-based alignment, or a task-specific metric) [11,12,13]. However, collapsing a inherently multi-objective problem into one dimension can cause several issues, such as (i) the metric gaming problem: improving the chosen proxy while qualitatively regressing elsewhere; (ii) unstable updates: high-variance advantages driven by noisy scorers; and (iii) brittle generalization: improvements that fail to transfer across benchmarks such as DrawBench [3] or GenEval [14].

**Figure 1:**
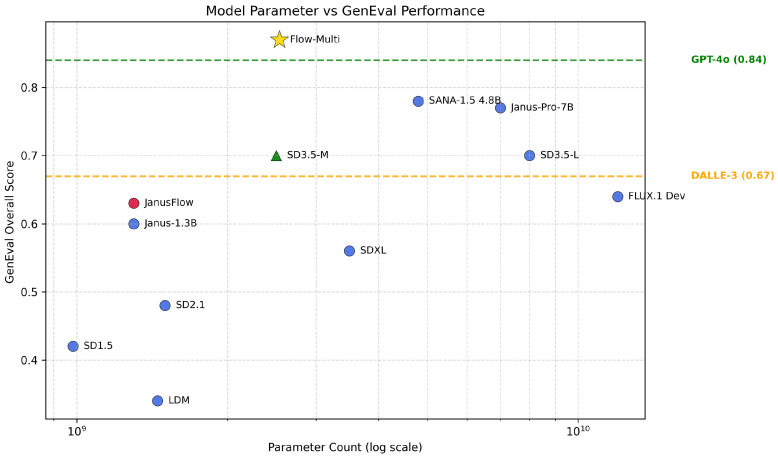
GenEval performance. The proposed Flow-Multi model demonstrates higher GenEval performance than GPT-4o and the baseline SD3.5-M, outperforming all other compared models.

To address these challenges, we propose Flow-Multi (Figure 1), a flow-matching, multi-reward reinforcement learning framework for T2I models. Building on group-relative policy optimization (GRPO) in a flow-matching sampler, Flow-Multi evaluates each generated sample with a vector of rewards that span four complementary dimensions: text–image alignment, human preference, aesthetic quality, and GenEval-style object/attribute correctness. Rather than simple averaging or handcrafted weight tuning, we treat the update as a multi-objective selection problem and use Pareto dominance to keep only non-dominated samples within each mini-batch. This method explicitly focuses on trade-off relationships, where a sample is retained if improving any of its rewards would worsen at least one other reward. In addition, we introduce advantage masking, a simple but effective mechanism that nullifies low-quality advantages so that policy gradients can be dominated by consistently good samples instead of noisy tails.

To train our proposed T2I framework, we operate based on the inner loop of sampling and learning. Our framework is also metric-agnostic, where reward heads can be swapped or extended without retooling the optimizer. Additionally, the flow-matching backbone keeps training stable and sample-efficient, while mini-batch Pareto selection provides a principled, computationally efficient approximation to multi-objective policy improvement. The experimental results show a more balanced performance profile across heterogeneous benchmarks, mitigating the typical “win one metric, lose two” failure mode of single-reward optimization.

## 2. Related Work

### 2.1. Diffusion and Flow-Matching Foundations

Diffusion models learn to reverse a Gaussian noising process and have become the dominant paradigm for high-fidelity image synthesis, with sampling implemented via discrete DDPM steps or continuous-time probability-flow ODE/SDE solvers [1,2,15]. Flow matching instead trains a continuous-time normalizing flow by directly matching the velocity field, enabling efficient deterministic sampling with far fewer steps while preserving quality [16,17]. Recent analyses connect diffusion and flow under a unified SDE/ODE view, clarifying when stochastic versus deterministic solvers are preferable and how model parameterizations transfer across the two families [10]. Building on this foundation, modern T2I systems often adopt flow-matching backbones for superior speed–quality trade-offs in both image and video generation.

### 2.2. Reinforcement Learning for T2I Alignment

Preference alignment for diffusion/flow generators has followed multiple tracks. Policy-gradient-style methods adapt RLHF ideas to T2I: DPO [18] directly fine-tunes diffusion policies from human preferences, while Diffusion-DPO [5] imports DPO as a simpler, value-free alternative to PPO-like RLHF [5]. Training-free alignment aims to steer preference at inference time without additional optimization [19]. More recently, group-relative policy optimization (GRPO [9])—a value-free policy gradient introduced for LLMs—has been instantiated on flow-matching generators: Flow-GRPO [10] integrates online RL into flows, and concurrent works (e.g., DanceGRPO [4], PREF-GRPO [20]) explore injecting stochasticity (converting the ODE to an SDE) or fitting pairwise preferences to stabilize advantages and mitigate reward hacking. While effective, single-reward training often exhibits metric gaming and trade-off regressions on unoptimized dimensions [21] (e.g., aesthetics vs. compositional correctness), motivating multi-reward formulations. Reinforcement learning has also been successfully applied to adaptive measurement and policy optimization problems in other domains, such as electromagnetic tomography, where RL enables efficient decision-making under complex constraints [22].

### 2.3. Multi-Objective Alignment and Reward Design

T2I post-training has been pursued via (1) direct scalar-reward fine-tuning [21], (2) reward backpropagation (AlignProp [13]), (3) DPO-style preference fitting [5,18], (4) PPO/GRPO policy gradients [9,23], and (5) training-free steering [19]. Many systems use linear scalarization of multiple rewards (e.g., CLIP alignment + aesthetics) with hand-tuned weights, as in Promptist [24] or differentiable reward formulations in DRaFT [6]. Parrot [25] explores multi-objective optimization by mapping language-encoded preference vectors to Pareto-optimal solutions, and model-averaging along the Pareto frontier has also been studied [8]. However, scalarization fixes a single operating point and is brittle when reward scales or reliabilities shift. Complementary to scalarization, process rewards seek finer credit assignment beyond sparse terminal signals: step-level PRMs and verification-guided supervision (ThinkPRM [26]) improve reasoning- or step-aware learning but carry substantial annotation/training cost. In diffusion/flow settings, step-aware preference models (e.g., LPO [27]) target both noisy and clean images; online surrogates (PRIME [28]) approximate process rewards from outcomes alone. Our perspective aligns with the latter: use multiple outcome rewards to reflect orthogonal desiderata (alignment, preference, aesthetics, compositionality) and select dominated samples via Pareto dominance, complemented by advantage masking to suppress low-quality gradients—thereby approximating multi-objective improvement without training explicit PRMs.

### 2.4. Benchmarks and Evaluation for T2I

Evaluation has evolved beyond perceptual fidelity to probe compositional reasoning, world knowledge, typography, and controllability. Datasets such as DrawBench [3] and GenEval [14] stress attribute binding, counts, spatial relations, and text rendering; newer suites (e.g., TIIF-Bench [29]) vary prompt length, typography, and style to test robustness under prompt perturbations, while UNIGENBENCH [30] expands coverage across fine-grained sub-criteria. Preference-oriented metrics (e.g., PickScore [31]), alignment metrics (e.g., CLIPScore [12]), and learned aesthetics predictors [11] complement task-specific pass/fail scores. Our experiments follow this trend, reporting multi-dimensional metrics (alignment, preference, aesthetics, GenEval correctness), which together reveal trade-offs that single metrics often conceal.

## 3. Method

### 3.1. Preliminaries: Group-Relative Policy Optimization for Flow-Based Models

We adopt the Rectified Flow framework [32] to define a generative process interpolating between a clean data sample $x_{0}∼X_{0}$ and a noise sample $x_{1}∼N\left(0,I\right)$ via a linear path $x_{t}=\left(1-t\right)x_{0}+tx_{1}$. A Transformer-based velocity field $v_{\theta }\left(x_{t},t\right)$ is trained to minimize the following flow-matching objective:

(1) $$L_{\mathrm{FM}}\left(\theta \right)=E_{t∼U\left[0,1\right],\;x_{0}∼X_{0},\;x_{1}∼X_{1}}\left[\;∥\left(x_{1}-x_{0}\right)-v_{\theta }\left(x_{t},t\right){∥}_{2}^{2}\;\right].$$

To leverage reinforcement learning (RL) for fine-tuning, we formulate the denoising process as a finite-horizon Markov Decision Process (MDP) where the state is $s_{t}=\left(x_{t},t,c\right)$, the action corresponds to the state update $a_{t}=\Delta x_{t}$, and the policy is induced by the flow model. Unlike standard deterministic ODE samplers, we adopt a Stochastic Differential Equation (SDE) formulation to enable exploration and define a valid probability density for the policy. The transition follows the Euler–Maruyama update as in

(2) $$x_{t+\Delta t}=x_{t}\;+\;\mu_{\theta }\left(x_{t},t,c\right)\;\Delta t\;+\;\sigma_{t}\sqrt{\Delta t}\;\epsilon ,\;\epsilon ∼N\left(0,I\right),$$

where the drift μ is parameterized as

(3) $$\mu_{\theta }\left(x_{t},t,c\right)=v_{\theta }\left(x_{t},t,c\right)\;+\;\frac{\sigma_{t}^{2}}{2t}(x_{t}+\left(1-t\right)\;v_{\theta }\left(x_{t},t,c\right)).$$

To enable exploration while approximately preserving the marginals of the corresponding deterministic flow ODE, we adopt an SDE formulation with an *x*-independent noise schedule $\sigma_{t}=a\sqrt{t/(1-t)}$. Under standard assumptions (e.g., small step size and matched drift–diffusion terms), this SDE is designed to induce marginal distributions that are consistent with those of the flow ODE, following prior formulations in flow-matching models. In our experiments, we fix the noise scale to a=0.7 and use S=20 sampling steps, resulting in a step size of Δt=1/20. Consequently, the policy $\pi_{\theta }\left(\cdot |s_{t}\right)$ becomes a Gaussian distribution $N(x_{t}+\mu_{\theta }\Delta t,\sigma_{t}^{2}\Delta tI)$, which facilitates gradient-based optimization.

Rather than introducing an explicit value function, we employ group-relative policy optimization (GRPO), which estimates advantages from relative comparisons within a sampled group. Specifically, for a given prompt $q_{i}$, we sample a group of *K* stochastic trajectories ${\left\{\tau_{i,k}\right\}}_{k=1}^{K}$ using the current policy. We define the outcome $o_{i,k}$ as the full sampling trajectory $\tau_{i,k}$, which consists of a sequence of state–action pairs generated by the SDE-based policy over *S* steps. For each trajectory, only the final generated image $x_{i,k}$ is evaluated to compute a terminal reward $R_{i,k}$. To reduce variance and avoid explicit-value-function estimation, we compute the group-relative advantage as follows:

(4) $$A_{i,k}=\frac{R_{i,k}-{\bar{R}}_{i}}{\max\;\left(\mathrm{Std}[R_{i,\cdot }],\;\epsilon \right)},\;{\bar{R}}_{i}=\frac{1}{K}\sum_{k=1}^{K}R_{i,k},$$

Based on these advantages, we define the likelihood ratio $\rho_{i,k}=\pi_{\theta }\left(o_{i,k}|q_{i}\right)/\pi_{\theta_{\mathrm{old}}}\left(o_{i,k}|q_{i}\right)$ and construct a PPO-style clipped surrogate objective as follows:

(5) $$L_{\mathrm{clip}}\left(\theta \right)=E_{i,k}\left[\min\;\left(\rho_{i,k}\;A_{i,k},\;\mathrm{clip}\left(\rho_{i,k},\;1-\epsilon_{\mathrm{clip}},\;1+\epsilon_{\mathrm{clip}}\right)\;A_{i,k}\right)\right].$$

In addition to the above surrogate loss, we incorporate a KL divergence penalty to a frozen reference policy $\pi_{\mathrm{ref}}$. While the general form relies on the density ratio $r\left(o\right)=\pi_{\mathrm{ref}}/\pi_{\theta }$, our diffusion-based policy with a fixed covariance schedule allows for a closed-form stepwise calculation:

(6) $$D_{\mathrm{KL}}\;\left(\pi_{\theta }\left(\cdot \;|\;s_{t}\right)\|\pi_{\mathrm{ref}}\left(\cdot \;|\;s_{t}\right)\right)=\frac{∥{\bar{x}}_{t+\Delta t,\theta }-{\bar{x}}_{t+\Delta t,\mathrm{ref}}{∥}_{2}^{2}}{2\;\sigma_{t}^{2}\Delta t},$$

where ${\bar{x}}_{t+\Delta t,\theta }$ and ${\bar{x}}_{t+\Delta t,\mathrm{ref}}$ are the mean updates of the active and reference policies, respectively. This simplifies to a Euclidean distance between drifts, directly regularizing the velocity field. Finally, combining these terms, the policy is updated by maximizing the objective:

(7) $$\max_{\theta }\;J_{\mathrm{GRPO}}\left(\theta \right)=L_{\mathrm{clip}}\left(\theta \right)\;-\;E_{i}\left[\beta \;D_{\mathrm{KL}}\;\left(\pi_{\theta }\left(\cdot \;|\;q_{i}\right)\|\pi_{\mathrm{ref}}\left(\cdot \;|\;q_{i}\right)\right)\right].$$

To implement this optimization efficiently, we utilize Low-Rank Adaptation (LoRA [33]). Instead of updating the full weights *W*, we freeze the pretrained parameters $W_{0}$ and train low-rank matrices *A* and *B* such that the effective weight becomes $W=W_{0}+BA$. This approach allows us to adapt the heavy text-to-image backbone to complex reward signals with minimal computational overhead.

### 3.2. Prompt Dataset and Grouping

For the input prompts, we adopt the GenEval [14] prompt set as our training/evaluation source. Specifically, training prompts are drawn exclusively from train_metadata.jsonl, while all evaluations are performed on the held-out prompts in test_metadata.jsonl, which are completely isolated from training. Each record in the metadata (train_metadata.jsonl) specifies a natural-language prompt and structured constraints such as object classes, counts, colors, and spatial relations (e.g., tag ∈ {counting, colors, position, color_attr}; fields include/exclude provide the target composition). We use the prompt string as input for generation, while the structured fields are used to compute the compositional reward.

For each prompt $q_{i}$, we generate a group of K=4 images ${\left\{x_{i,k}\right\}}_{k=1}^{K}$ using our diffusion policy (Section 3.3). In our default setting, each epoch uses P=48 prompts (mini-batches), yielding N=P×K=192 images per epoch. Grouping by prompt is essential because both our advantage estimation and our Pareto selection are done within the set ${\left\{x_{i,k}\right\}}_{k=1}^{K}$ for a fixed $q_{i}$.

### 3.3. Flow-Matching Sampling with GRPO

We use a Stable Diffusion-3.5-medium model and perform sampling with a flow-matching scheduler. Unless otherwise noted, we use S=10 sampling steps for training (exploration), $S_{\mathrm{eval}}\;=\;40$ for evaluation, classifier-free guidance scale =4.5, and 512 × 512 resolution. For a prompt $q_{i}$, the policy $\pi_{\theta }\left(\cdot |q_{i}\right)$ induces a per-step conditional Gaussian transition; we log the per-step log-likelihood ratio to construct the PPO-style clipped objective in GRPO (Section 3.7).

### 3.4. Reward Suite

Each generated image $x_{i,k}$ is evaluated by four metrics, which together form a 4D reward vector as in

$$R\left(x_{i,k}\right)=\left[r_{i,k}^{\mathrm{aesth}},\;r_{i,k}^{\mathrm{pref}},\;r_{i,k}^{\mathrm{align}},\;r_{i,k}^{\mathrm{comp}}\right]\in R^{4}.$$

Concretely,

- **Aesthetic Quality** ($r^{\mathrm{aesth}}$): A learned aesthetic predictor score.
- **Human Preference** ($r^{\mathrm{pref}}$): PickScore (CLIP ViT-H-14 with preference head).
- **Text–Image Alignment** ($r^{\mathrm{align}}$): CLIPScore between the prompt and the image.
- **Compositional Compliance** ($r^{\mathrm{comp}}$): GenEval-style object/attribute/position/count correctness, computed using the metadata (include/exclude) with a pretrained detector.

Stacking $R(x_{i,k})$ over all *N* images yields a matrix $R\in R^{N\times 4}$ per epoch. We note that the GenEval-style compositional correctness used as $r^{\mathrm{comp}}$ is computed using the same detector-based evaluation pipeline as the GenEval Overall metric reported in evaluation. In this work, $r^{\mathrm{comp}}$ is used as a training-time diagnostic signal for enforcing compositional constraints, rather than as an independent generalization objective.

### 3.5. Group-Wise Pareto Non-Dominated Selection

Within each prompt group *i*, we consider the *K* reward vectors ${\left\{R\left(x_{i,k}\right)\right\}}_{k=1}^{K}$. A sample $x_{i,a}$ dominates $x_{i,b}$ (denoted $x_{i,b}≺x_{i,a}$) if it is no worse on all metrics and strictly better on at least one:

$$R\left(x_{i,a}\right)⪰R\left(x_{i,b}\right)\;⟺\;\left(R_{d}\left(x_{i,a}\right)\geq R_{d}\left(x_{i,b}\right)\;\forall d\right)\;\wedge \;\left(\exists d:\;R_{d}\left(x_{i,a}\right)>R_{d}\left(x_{i,b}\right)\right),$$

where we mark each non-dominated sample with a binary mask as in

$$M_{i,k}=\left\{\begin{array}{cc} 1, & \mathrm{if}\;x_{i,k}\;\mathrm{is}\;\mathrm{non}\text{-}\mathrm{dominated}\;\mathrm{within}\;\mathrm{group}\;i,\\ 0, & \mathrm{otherwise}. \end{array}\right.$$

Consequently, only the survivors $\left\{k:\;M_{i,k}=1\right\}$ enter the subsequent statistics and policy update. This batchwise Pareto filtering preserves diverse trade-offs among the four objectives while removing samples strictly inferior to others under the same prompt.

### 3.6. Scalarization and Survivor-Only Advantage

After group-wise Pareto filtering (Section 3.5), we compute a single scalar reward per image as a fixed weighted sum of the four metrics. This filtering step is crucial; unlike naive scalarization, which might favor samples that exploit a single reward while degrading others (reward hacking), Pareto selection ensures that only solutions offering valid trade-offs across all objectives contribute to the policy update. Importantly, the weighted aggregation is applied only after Pareto filtering and does not affect the construction of the Pareto set itself. Let $w=w^{\mathrm{aesth}},w^{\mathrm{pref}},w^{\mathrm{align}},w^{\mathrm{comp}}$ denote the reward weights specified in the configuration file (config.reward_fn); by default, we set $w_{d}=\frac{1}{4}$. For each image $x_{i,k}$, we define the combined reward $r_{i,k}^{\mathrm{comb}}$ as follows:

$$r_{i,k}^{\mathrm{comb}}\;=\;\sum_{d=1}^{4}w^{\left(d\right)}\;r_{i,k}^{\left(d\right)},$$

which matches the avg in our implementation. Let $M_{i,k}\in \left\{0,1\right\}$ denote the Pareto non-dominance mask within each prompt group (Section 3.5), and let $𝒮=\{\left(i,k\right):M_{i,k}=\;1\;\wedge \;\mathrm{valid}\left(i,k\right)\}$ be the set of surviving, valid samples across the whole distributed mini-epoch (all processes). If 𝒮 is empty, we fall back to a single top-1 valid sample by $r^{\mathrm{comb}}$ to avoid degeneracy. Then, we compute global survivor-only statistics as follows:

$$\mu_{𝒮}\;=\;\frac{1}{\left|𝒮\right|}\sum_{(i,k)\in 𝒮}r_{i,k}^{\mathrm{comb}},\;\sigma_{𝒮}\;=\;\max\;\left(\mathrm{Std}\{r_{i,k}^{\mathrm{comb}}:\left(i,k\right)\in 𝒮\},\;\epsilon \right),$$

and define the advantage $A_{i,k}$ using the above statistics as in

$$A_{i,k}\;=\;\frac{r_{i,k}^{\mathrm{comb}}-\mu_{𝒮}}{\sigma_{𝒮}}\;\cdot \;M_{i,k}.$$

Thus, dominated or invalid samples ($M_{i,k}=0$) contribute zero advantage. We do not compute per-step advantages.For terminal-only rewards, we use a single scalar $A_{i,k}$ per image and apply it to all stepwise log-prob terms; equivalently, we set $A_{i,k,t}:=A_{i,k}$ for notation/shape matching only.

### 3.7. GRPO with Pareto Masking

Reusing the definitions from Section 3.1, where $o_{i,k}$ denotes the full trajectory, the likelihood ratio $\rho_{i,k}$ represents the cumulative probability ratio over the sampling steps:

(8) $$\rho_{i,k}=\frac{\pi_{\theta }\left(o_{i,k}|q_{i}\right)}{\pi_{\theta_{\mathrm{old}}}\left(o_{i,k}|q_{i}\right)}=\prod_{t=0}^{S-1}\frac{\pi_{\theta }\left(x_{t+\Delta t}|x_{t},q_{i}\right)}{\pi_{\theta_{\mathrm{old}}}\left(x_{t+\Delta t}|x_{t},q_{i}\right)}$$

so we can define the PPO-style clipped surrogate objective with Pareto masking as in

(9) $$L_{\mathrm{clip}}^{\mathrm{mask}}\left(\theta \right)=E_{i}\left[\frac{1}{\sum_{k}M_{i,k}}\sum_{k=1}^{K}\min\;\left(\rho_{i,k}\;A_{i,k}\;M_{i,k},\;\mathrm{clip}\left(\rho_{i,k},1-\epsilon_{\mathrm{clip}},1+\epsilon_{\mathrm{clip}}\right)\;A_{i,k}\;M_{i,k}\right)\right].$$

**Figure 2:**
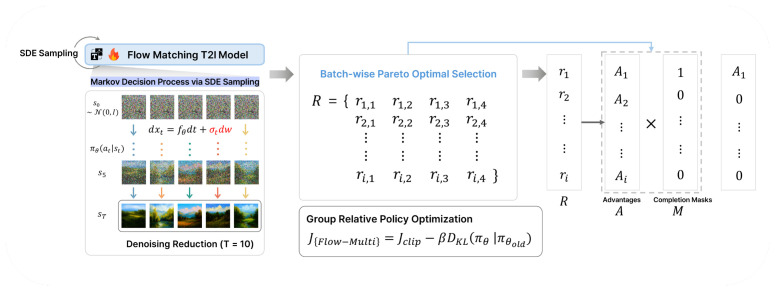
Overview of Flow-Multi, a multi-reward reinforcement learning framework incorporating Pareto optimization and advantage masking.

Finally, we also include a trust-region penalty toward a frozen reference policy $\pi_{\mathrm{ref}}$ as in

(10) $$\max_{\theta }\;\;J_{\mathrm{GRPO}}\left(\theta \right)=L_{\mathrm{clip}}^{\mathrm{mask}}\left(\theta \right)\;-\;E_{i}\left[\beta \;D_{\mathrm{KL}}\;\left(\pi_{\theta }\left(\cdot \;|\;q_{i}\right)\;∥\;\pi_{\mathrm{ref}}\left(\cdot \;|\;q_{i}\right)\right)\right],$$

where $D_{\mathrm{KL}}$ is computed from log-likelihoods (or, under equal-covariance Gaussian steps, via a per-step closed form). In practice, we optimize $J_{\mathrm{GRPO}}$ with $\epsilon_{\mathrm{clip}}$ and β tuned to balance stability and exploration. For our implementation, we perform the following steps. At each epoch we (i) sample N=192 images (48 prompts × 4 images), (ii) compute the four rewards and build $R\in R^{N\times 4}$, (iii) apply group-wise Pareto to obtain $\left\{M_{i,k}\right\}$, (iv) compute survivor-only normalized advantages, and (v) update the policy with the masked GRPO objective (9) and (10). The Pareto keep ratio ($\sum_{i,k}M_{i,k}/N$) is tracked to monitor how selective the filtering is during training. The overall step-by-step training algorithm for our framework is shown in Algorithm 1. The overview for our proposed framework is shown in Figure 2.
**Algorithm 1** Flow-Multi with Pareto Masking (mini-batch)**Require:** $\pi_{\theta }$: Online policy model (initialized from pretrained weights) 
$\pi_{\mathrm{ref}}$: Reference model 
Q: prompt batch 
*K*: group size (default 4) 
$\sigma_{t}=a\sqrt{t/(1-t)}$: noise schedule 
$\mu_{\theta }$ (Equation (3): drift; β: KL weight; LoRA rank *r*; reward weights $w\in R^{4}$ (default $w_{d}\;=\;\frac{1}{4}$))
**Ensure:** updated policy $\pi_{\theta }$ (LoRA-only updates)
  1: Initialize policy $\pi_{\theta }$ (LoRA on Q/K/V/O); set reference $\pi_{\mathrm{ref}}\leftarrow \mathrm{stopgrad}\left(\pi_{\theta }\right)$  2: **for** epoch =1,… **do**
  3:       Sample a mini-batch of prompts ${\left\{q_{i}\right\}}_{i=1}^{P}$ from Q
  4:       **for** each prompt $q_{i}$ **do**
  5:             **Sampling (SDE rollout)**: generate *K* images ${\left\{x_{i,k}\right\}}_{k=1}^{K}$ using (2)
  6:             **Reward vector** for each $x_{i,k}$: $R\left(x_{i,k}\right)=\left(r_{\mathrm{aesth}},r_{\mathrm{pref}},r_{\mathrm{align}},r_{\mathrm{comp}}\right)$
  7:             **Group-wise Pareto** non-dominated mask $M_{i,k}\in \left\{0,1\right\}$                 (Section 3.5)
  8:             **Scalarization** $r_{i,k}^{\mathrm{comb}}=\sum_{d=1}^{4}w^{\left(d\right)}r_{i,k}^{\left(d\right)}$                          (Section 3.6)
  9:       **end for**
10:       Gather survivors $S=\{\left(i,k\right):M_{i,k}=1\wedge \mathrm{valid}\left(i,k\right)\}$ across devices; if S=∅ then fallback to top-1 by $r^{\mathrm{comb}}$
11:       Compute survivors-only stats $\mu_{S}$, $\sigma_{S}$
12:       **Advantage (masked)** $A_{i,k}=\frac{r_{i,k}^{\mathrm{comb}}-\mu_{S}}{\sigma_{S}}\cdot M_{i,k}$
13:       Compute likelihood ratios $\rho_{i,k}=\frac{\pi_{\theta }\left(o_{i,k}|q_{i}\right)}{\pi_{\theta_{\mathrm{old}}}\left(o_{i,k}|q_{i}\right)}$
14:       **PPO-style objective with Pareto mask**                   (Equation (9), Section 3.7)
15:       **KL penalty to reference** (stepwise or sequence):               (Equation (10), Section 3.7)
16:       Update $\theta \leftarrow \theta +\eta \;\nabla_{\theta }J_{\mathrm{GRPO}}\left(\theta \right)$                          ▹ LoRA params only
17:       **Logging:** Pareto keep ratio $\left(\sum_{i,k}M_{i,k}/\left(P\;\cdot \;K\right)\right)$, each reward mean/var, *A* stats, KL, loss terms, learning curves
18:       Periodically refresh reference $\pi_{\mathrm{ref}}\leftarrow \mathrm{stopgrad}\left(\pi_{\theta }\right)$
19: **end for**


## 4. Experiments

To quantitatively evaluate our proposed method, the GenEval [14] benchmark is used, which evaluates compositional reasoning across diverse tasks: Single Obj. (single-target accuracy), Two Obj. (multi-object co-occurrence), Counting (numerosity), Colors (attribute correctness), Position (spatial reasoning), and Attr. Binding (object–attribute association). The Overall score represents the arithmetic mean of these sub-tasks. To ensure a fair comparison, all open-source baseline models and our proposed method were evaluated under a strictly unified experimental environment with identical hyperparameters. However, due to the proprietary nature and API-only access of DALL·E 2/3 and GPT-4o, local reproduction under these identical conditions is infeasible. Therefore, their results are cited directly from official sources. Notably, for Flow-GRPO, we report results reproduced under our setup; these differ from the originally reported Overall score.

As shown in Table 1, our proposed method, Flow-Multi, substantially outperforms the base SD3.5-M model, raising the Overall score from 0.70 to 0.87 (approximately +26%). Furthermore, Flow-Multi surpasses the reproduced Flow-GRPO baseline (0.72 Overall) while maintaining consistent strength across all sub-tasks. Specifically, Flow-Multi achieves near-perfect accuracy in multi-object composition (Two Obj.: 0.99) and demonstrates significant gains in Counting (0.88), Position (0.80), and Attr. Binding (0.80). These results highlight the efficacy of multi-reward reinforcement learning in enhancing text-to-image alignment beyond single-objective optimization.

Table 2 expands this analysis to include image quality and human preference. We report CLIP (semantic similarity), Aesthetic (perceptual quality), and DeQA (visual fidelity/artifacts), alongside human preference proxies ImageReward, PickScore, and the aggregated UnifiedReward. A critical analysis of Table 2 reveals that Flow-Multi achieves a superior trade-off between alignment and quality compared to Flow-GRPO. While Flow-GRPO improves alignment over SD3.5-M, it suffers from a degradation in visual quality, evidenced by a drop in Aesthetic score from 5.80 to 5.63. This suggests that optimizing for a single alignment-oriented reward can harm perceptual fidelity. In contrast, Flow-Multi not only secures a higher GenEval score (0.87) but also preserves visual realism, recovering the Aesthetic score to 5.86 and improving DeQA to 4.15—outperforming Flow-GRPO by +0.23 and +0.22, respectively. This demonstrates that multi-reward optimization effectively mitigates the alignment–quality trade-off.

**Figure 3:**
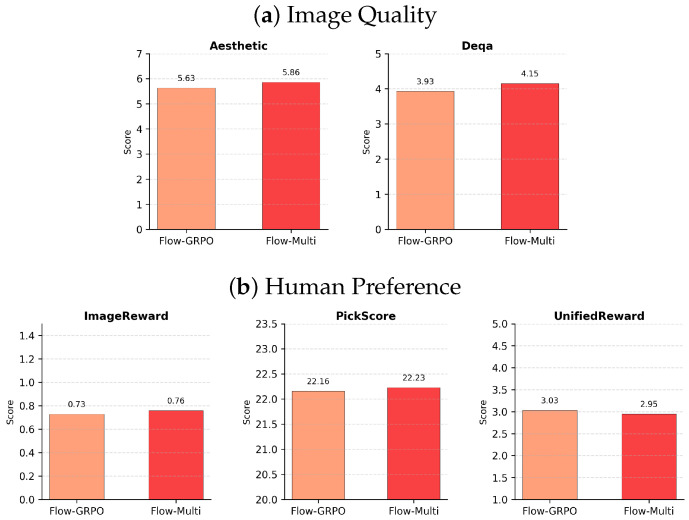
(**a**) Image quality metrics (Aesthetic, DeQA) remain stable across models. (**b**) Human preference metrics (ImageReward, PickScore, UnifiedReward) improve after flow fine-tuning.

We compare the perceptual quality and human preference metrics of Flow-GRPO and Flow-Multi, where the results are shown in Figure 3. As observed in (a), Flow-GRPO tends to sacrifice image quality (lower Aesthetic and DeQA scores) in exchange for strict text alignment. In contrast, Flow-Multi effectively mitigates this trade-off, achieving higher Aesthetic and DeQA scores. This improvement extends to (b) human preference metrics; Flow-Multi outperforms Flow-GRPO in both ImageReward and PickScore. Although the UnifiedReward is comparable, the consistent gains in aesthetic and preference scores confirm that Flow-Multi generates more visually appealing images while maintaining alignment.

**Figure 4:**
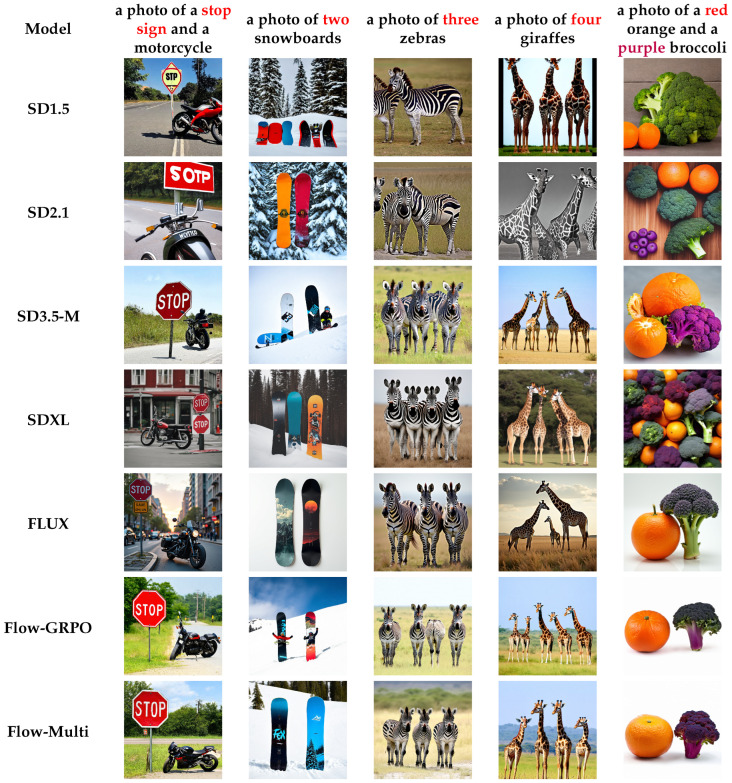
Compared to other models, Flow-Multi outperforms SD-based baselines in color accuracy, counting, and aesthetic quality.

**Figure 5:**
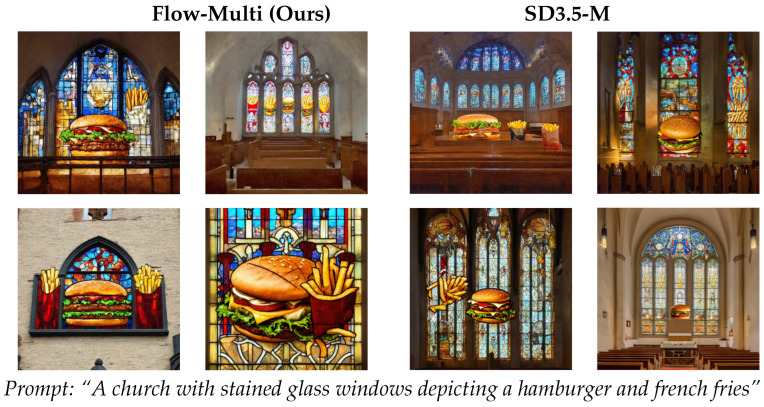
Qualitative comparison between Flow-Multi and SD3.5-M on DrawBench. Each prompt block shows four sampled generations from each model. Flow-Multi demonstrates improved text–image alignment and compositional consistency, while preserving visual fidelity across diverse samples.

We show the qualitative examples of generations on compositional prompts in Figure 4 and Figure 5. Flow-Multi accurately aligns multiple objects and reliably handles counting prompts (“two snowboards”), generating the correct number of distinct, non-overlapping instances. It also preserves object identity in challenging cases such as zebras and giraffes, and achieves strong color–attribute consistency, showing improved compositional fidelity over prior SD-based models.

Table 3 presents a comprehensive comparison of computational complexity and generation performance. To ensure a fair evaluation, we measured the inference latency and FLOPs of all models in an identical environment using a single NVIDIA RTX-A6000. Notably, Flow-Multi achieves a significant improvement in GenEval score (0.70 → 0.87) over its backbone, SD 3.5-Medium, while maintaining identical parameters and inference costs. This demonstrates that our framework enhances alignment without any additional computational overhead. Furthermore, compared to resource-intensive models like SD 3.5-Large and FLUX.1 Dev, Flow-Multi exhibits superior efficiency, delivering competitive performance with significantly lower resource requirements. While autoregressive models like Janus show lower FLOPs, their latency is practically constrained by memory bandwidth.

## 5. Conclusions

In this paper, we identify fundamental limitations of the conventional reinforcement learning (RL) approach for text-to-image (T2I) generation, where collapsing multi-dimensional human preferences into a single scalar reward leads to brittle generalization and misalignment. Such reward aggregation obscures inherent trade-offs among quality dimensions, resulting in unstable optimization and biased alignment.

To address this issue, we proposed Flow-Multi, a multi-reward RL framework based on flow-matching GRPO. Flow-Multi combines vector-valued rewards, group-wise Pareto non-dominated sample selection, and advantage masking, enabling stable policy updates and balanced optimization across multiple objectives without heuristic tuning.

Experiments on the GenEval benchmark demonstrate clear performance gains: Flow-Multi improves SD3.5-M from 0.70 to 0.87, outperforming the single-reward Flow-GRPO baseline (0.72). In addition to higher aggregate scores, Flow-Multi achieves consistent improvements in spatial reasoning and attribute binding, with human evaluations confirming superior perceptual alignment.

Overall, we establish a principled framework for multi-objective alignment in generative modeling. By reframing T2I alignment as a multi-objective optimization problem, Flow-Multi provides a scalable alternative to single-reward RL and can offer a strong baseline for aligning future large-scale multimodal generative models.

## Figures and Tables

**Table 1:** GenEval image generation benchmark. Best and second-best scores are highlighted in blue and green, respectively.

Model	Overall	Single Obj.	Two Obj.	Counting	Colors	Position	Attr. Binding
*Diffusion Models*							
LDM [34]	0.34	0.90	0.26	0.19	0.66	0.01	0.04
SD1.5 [34]	0.42	0.95	0.37	0.35	0.75	0.03	0.06
SD2.1 [34]	0.48	0.98	0.45	0.40	0.82	0.08	0.14
SD-XL [35]	0.56	0.98	0.75	0.43	0.88	0.13	0.21
DALLE-2 [36]	0.52	0.94	0.66	0.49	0.77	0.10	0.19
DALLE-3 [37]	0.67	0.96	0.87	0.47	0.83	0.43	0.45
*Autoregressive Models*							
Janus-1.3B [38]	0.60	0.96	0.62	0.27	0.82	0.48	0.46
Janus-Pro-7B [39]	0.77	0.97	0.87	0.55	0.88	0.73	0.62
GPT-4o [40]	0.84	0.99	0.92	0.85	0.92	0.75	0.61
*Flow-Matching Models*							
FLUX.1 Dev [41]	0.64	0.98	0.79	0.75	0.77	0.20	0.39
SD3.5-L [42]	0.71	0.99	0.90	0.69	0.84	0.26	0.60
SANA-1.5 4.8B [43]	0.78	0.99	0.91	0.80	0.87	0.60	0.54
SD3.5-M [42]	0.70	0.99	0.89	0.69	0.82	0.27	0.55
Flow-GRPO [10]	0.72	1.00	0.90	0.73	0.82	0.30	0.59
Flow-Multi (Ours)	0.87	0.99	0.99	0.88	0.81	0.80	0.80

**Table 2:** Task performance and image-quality results on compositional generation, text rendering, and human preference benchmarks, evaluated using CLIPScore, ImageReward, and UnifiedReward.

Model	Task Metric		Image Quality		Preference Score		
	GenEval [14]	CLIP [12]	Aesthetic [11]	DeQA [44]	ImgRwd [45]	PickScore [31]	UniRwd [30]
*Diffusion Models*							
LDM [34]	0.34	0.19	5.06	3.35	−1.95	19.17	1.36
SD1.5 [34]	0.42	0.22	5.21	3.58	−1.48	19.78	1.58
SD2.1 [34]	0.48	0.24	5.45	3.66	−0.73	20.55	1.98
SD-XL [35]	0.56	0.26	5.88	3.92	0.15	21.78	2.61
*Autoregressive Models*							
Janus-1.3B [38]	0.60	0.21	5.28	2.87	−1.08	19.86	1.50
Janus-Pro-7B [39]	0.77	0.27	5.99	3.51	0.77	22.00	2.67
*Flow-Matching Models*							
FLUX.1 Dev [41]	0.64	0.27	6.26	4.35	1.03	22.87	3.34
SD3.5-L [42]	0.70	0.28	5.96	4.18	0.95	22.77	3.24
SANA-1.5 4.8B [43]	0.78	0.27	6.15	4.07	1.06	22.73	3.21
SD3.5-M [42]	0.70	0.28	5.80	4.14	0.77	22.32	3.02
Flow-GRPO [10]	0.72	0.28	5.63	3.93	0.73	22.16	3.03
Flow-Multi	0.87	0.28	5.86	4.15	0.76	22.23	2.95
Δ	+0.15	0.00	+0.23	+0.22	+0.03	+0.07	−0.08

Δ denotes the performance difference between Flow-Multi and Flow-GRPO.

**Table 3:** Comparison of computational complexity and performance. We compare the model size, inference cost (FLOPs), and inference latency against GenEval performance. All measurements were conducted in an identical environment. And - denotes metrics that were not obtainable due to hardware memory constraints during measurement.

Model	Params (B)	Inference TFLOPs (T)	Inference Time (s)	GenEval Score
LDM	0.86	135.10	8.40	0.34
SD 1.5	0.86	135.10	8.40	0.42
SD 2.1	0.87	135.14	6.72	0.48
SDXL	2.57	299.02	9.85	0.56
Janus-1.3B	2.09	1.66	13.74	0.60
Janus-Pro-7B	7.42	7.48	16.35	0.77
SANA-1.5	4.72	436.28	21.49	0.78
SD 3.5-L	8.06	1146.22	40.79	0.70
FLUX.1 Dev	11.90	-	48.06	0.64
SD 3.5-M	2.24	339.05	16.86	0.70
Flow-GRPO	2.24	339.05	16.83	0.72
Flow-Multi (Ours)	2.24	339.05	16.87	0.87

## Data Availability

The source code supporting the findings of this study is publicly available at https://github.com/2JAE22/Flow-Multi. The datasets used and generated during the current study are included in the article. Additional experimental details and configurations are available from the corresponding author upon reasonable request.
